# Semi‐Independent Control of Stability and Mobility in DNA Condensates

**DOI:** 10.1002/cbic.202500927

**Published:** 2026-02-23

**Authors:** Naoki Yoshida, Kei Goraku, Ryohei Furuichi, Mitsunori Takano, Yusuke Sato, Masahiro Takinoue

**Affiliations:** ^1^ Department of Life Science and Technology Institute of Science Tokyo Yokohama Japan; ^2^ Department of Computer Science Institute of Science Tokyo Yokohama Japan; ^3^ Department of Pure and Applied Physics Waseda University Tokyo Japan; ^4^ Department of Intelligent and Control Systems Kyushu Institute of Technology Fukuoka Japan; ^5^ Research Center for Autonomous Systems Materialogy (ASMat) Institute of Integrated Research Institute of Science Tokyo Yokohama Japan; ^6^ Laboratory for Chemistry and Life Science Institute of Integrated Research Institute of Science Tokyo Yokohama Japan

**Keywords:** bottom‐up synthetic biology, DNA condensates, DNA droplets, DNA nanotechnology, liquid–liquid phase separation

## Abstract

Liquid‐like biomolecular condensates possess unique physical properties that are essential for cellular functions and artificial cell engineering. However, increasing the thermal stability of these condensates typically reduces their liquid‐like dynamics. Here, we report a strategy to semi‐independently control the thermal stability and dynamic properties of DNA droplets formed from six‐branched DNA nanostructures (S‐motifs). We designed S‐motifs containing four fixed sticky ends (SEs) composed of 4 nucleotides (nt) and two variable‐length SEs of 0–20 nt in length. Extending the length of the two variable SEs increased the phase‐separation temperature (*T*_p_) of the droplets. Within a specific range (4–12 nt), where the melting temperature (*T*_m_) of the variable SEs remained below *T*_p_, the droplets maintained their fusion dynamics and internal mobility despite increased thermal stability. In contrast, when the variable SE length was 16–20 nt and *T*_m_ exceeded *T*_p_, the dynamic behaviors were inhibited because of stable polymerization. These findings demonstrate that the partial modification of the SE binding strength enables the tuning of thermal stability without sacrificing liquid fluidity, providing a valuable design principle for developing functional DNA‐based artificial cells and molecular robots.

## Graphical abstract

This article reports a strategy to semi‐independently control thermal stability and dynamics of DNA droplets composed of branched DNA nanostructures. By extending two variable sticky ends (SEs) while maintaining the others, the thermal stability increased without significantly reducing liquid‐like fusion or internal mobility. This demonstrates that partial SE modification enables stability tuning without sacrificing droplet fluidity for artificial cell engineering.

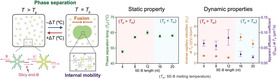

## 1 Introduction

Liquid‐like biomolecular condensates (biomolecular droplets), formed through liquid–liquid phase separation, have attracted significant interest due to their crucial role in various intracellular processes [1, 2]. These condensates exhibit unique functions, such as activating/inhibiting molecular reactions by localizing biomolecules [2], which in turn influence cellular functions and diseases [3, 4, 5, 6]. These essential functions are realized by their physical properties that combine both gel‐like thermodynamic stability and liquid‐like fluidity [2, 4]. These physical properties have also inspired the creation of artificial cells and organelles with novel functions beyond cellular systems [7, 8]. Consequently, understanding and engineering the physical properties of biomolecular droplets is becoming increasingly important.

**FIGURE 1:**
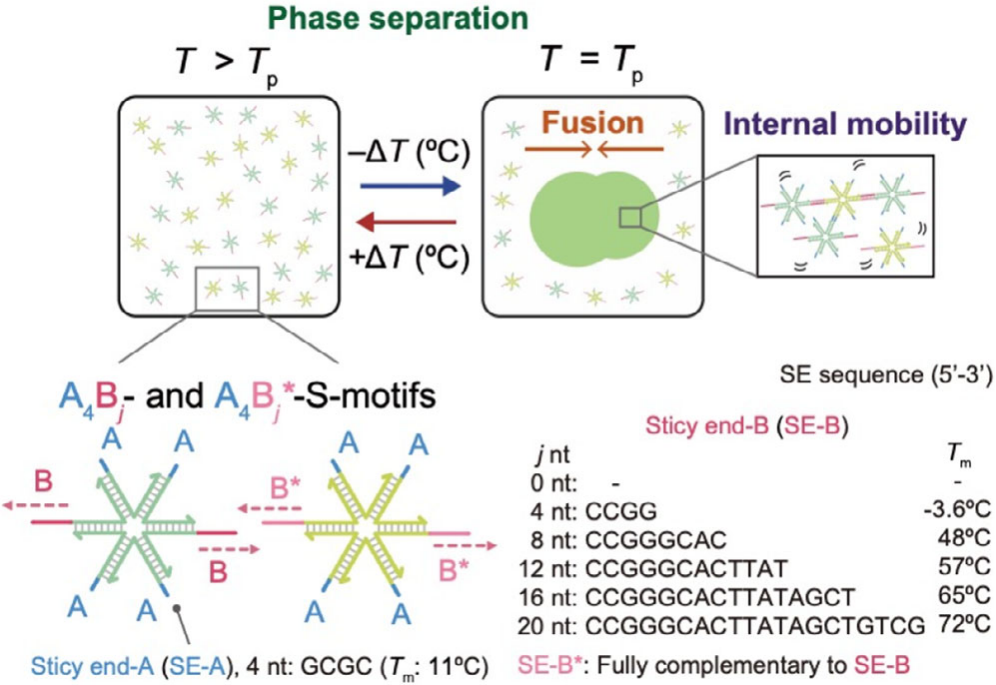
Phase separation, fusion, and internal mobility of DNA droplets. DNA droplets were formed through the self‐assembly of six‐branched DNA nanostructures (S‐motifs) via SE interactions. The S‐motifs have two types of sticky ends, SE‐A_4_ and SE‐B_*j*_, and are named A_4_B_*j*_‐ and A_4_B_j_^*^‐S‐motifs. *j* represents the nucleotide (nt) length of SE‐B. SE‐A is a self‐complementary sequence, and SE‐B^*^ is complementary to SE‐B. The melting temperatures (*T*_m_) of the SE were calculated using the nearest‐neighbor model [24] under the following conditions: 10 µM SE‐A, 5 µM SE‐B, and 350 mM NaCl.

DNA‐based synthetic biomolecular condensates have attracted significant interest because of their ability to form highly programmable structures [9, 10, 11]. The development of DNA condensates has mainly stemmed from branched DNA nanostructures (also known as branched DNA motifs or DNA nanostars) [12, 13] capable of forming networks [14]. This has led to the development of various DNA‐based materials, including dendrimers [15, 16], nano/microgels [17, 18], hydrogels [19], crystals [20], and more recently, droplets (condensates) [21, 22, 23]. DNA droplets, formed through the self‐assembly of DNA motifs via sticky‐end (SE) hybridization (Figure 1), offer programmable static and dynamic physical properties by designing the number of SEs [21, 25, 26], the length [27] and flexibility [22, 28] of each branched arm, and the binding strength of the SEs [25, 29]. DNA droplets composed of branched DNA motifs have been engineered to emulate cell‐like behaviors, such as growth [27], division [25, 30, 31], deformation [32, 33, 34], pattern formation [35, 36], and molecular communication [37]. They also facilitate various bioapplications, including controlled/triggered drug release [18, 38], biomolecular sensing/computing [39, 40], antimicrobial [41]/anticancer [42], and synthetic organelles engineering [43, 44].

One of the static properties of DNA droplets is thermal stability; the higher the droplet stability, the easier it is to manipulate. However, enhancing droplet stability often involves a trade‐off: as stability increases, their dynamic, liquid‐like behavior decreases. For instance, we demonstrated that extending SE length (i.e., stabilizing SE binding) of a DNA motif raised the phase‐separation temperature of DNA droplets (*T*_p_) but slowed the fusion dynamics at *T*_p_ [29]. Increasing the number of branch arms with SE enhanced both *T*_p_ and fusion dynamics at *T*_p_, whereas it reduced the internal mobility of the DNA motifs [25, 29]. SE hybridization can govern both *T*_p_ and internal mobility at *T*_p_. An increase in the number of SEs on a motif can lead to a higher collision frequency of SEs per motif, facilitating the formation of SE duplexes. This process could raise *T*_p_ while reducing internal mobility. In bulk systems, the number of SEs influenced *T*_p_ and critical dynamics [21], as well as elastic properties in the gel state [26]. Therefore, designing thermal stability independently of dynamic properties remains challenging.

In this study, we designed two types of six‐branched DNA nanostructures (S‐motifs) with SE‐A_4_ and SE‐B_*j*_ (*j* = 0,4,8,12,16, and 20), referred to as A_4_B_*j*_‐ and A_4_B_j_*‐S‐motifs (Figure 1), to semi‐independently control the thermal stability (related to the phase‐separation temperature, *T*_p_) while maintaining the dynamic behaviors of DNA droplets at *T*_p_, such as the fusion behavior of DNA droplets and internal mobility of S‐motifs. Here, the subscripts of SE‐A and SE‐B indicate their lengths, and B_j_* represents the complementary sequence of B_*j*_. We hypothesized that the thermal stability could be tuned independently of the dynamic behaviors by partially altering the length of only two SE‐Bs of the six SEs, while keeping the other four SE‐As unchanged. Because a 4‐nt SE has low binding stability, the 4‐nt sequence was used for SE‐A to isolate the effects of SE‐B. In this study, we investigated the effects of this partial change on the *T*_p_ and on dynamic properties of DNA droplets at *T*_p_.

## 2 Results and Discussion

**FIGURE 2:**
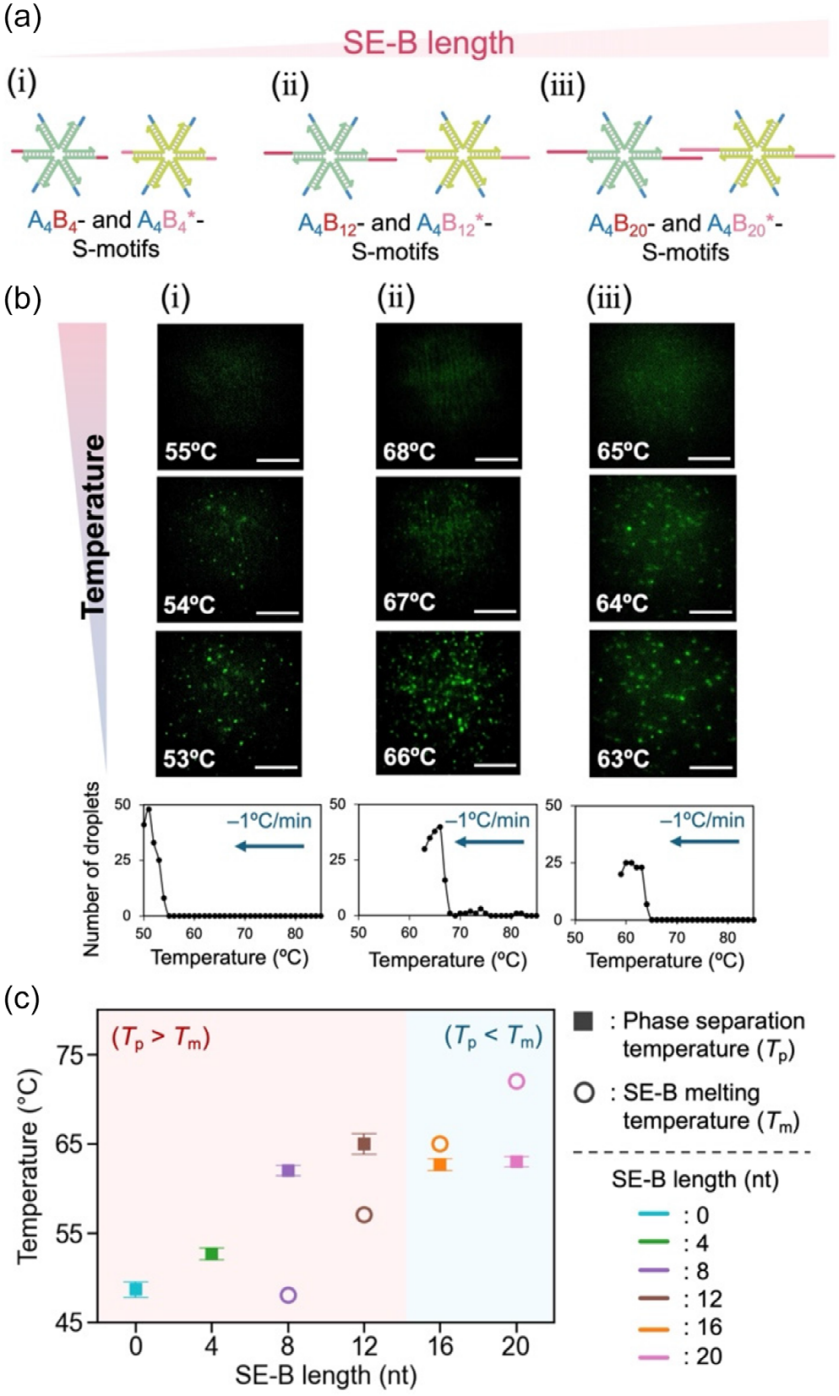
SE‐B length dependence of DNA droplet phase separation. (a) S‐motif design with SE‐A fixed and SE‐B selectively elongated. (b) Representative sequential images composed of (i) A_4_B_4_‐ and A_4_B_4_^*^‐S‐motifs, (ii) A_4_B_12_‐ and A_4_B_12_^*^‐S‐motifs, and (iii) A_4_B_20_‐ and A_4_B_20_^*^‐S‐motifs**.** Scale bars: 40 μm. (c) SE‐B sequence‐dependent *T*_p_ and SE‐B *T*_m_. *T*_p_ was determined based on the temperature‐dependent number of droplets (b, bottom). Error bars indicate standard error (*n* = 3).

Figure 2 shows the investigation of the thermal stability of DNA droplets, focusing on the length of the two SE‐Bs while maintaining the four SE‐As (Figure 2a). The formation of DNA droplets through liquid–liquid phase separation was examined using confocal laser scanning microscopy and analyzed using ImageJ. Fluorescently labeled (6‐carboxyfluorescein, FAM) A_4_B_*j*_‐S‐motifs were used at 20% molar concentration in place of nonlabelled A_4_B_*j*_‐S‐motifs for visualization (Figure S1a). The DNA droplets composed of A_4_B_4_‐ and A_4_B_4_*‐S‐motifs formed below 53°C as the temperature decreased from 85°C at −1°C/min (Figure 2a‐i,b‐i (top)). The number of DNA droplets was counted in the microscope images as the temperature decreased (Figure 2b, bottom), and *T*_p_ was defined as the temperature at which the number reached five droplets. In contrast, DNA droplets composed of A_4_B_12_‐ and A_4_B_12_*‐S‐motifs (Figure 2a‐ii,b‐ii (top)) and those composed of A_4_B_20_‐ and A_4_B_20_*‐S‐motifs (Figure 2a‐iii,b‐iii (top)) formed below 66 and 63°C, respectively.

The relationship between *T*_p_ and SE‐B length is shown in Figure 2c, derived from the data in Figures 2b and S1. As SE‐B_*j*_ (*j* = 0–12) was extended, *T*_p_ increased (Figures 2c and S1). Beyond 16 nt, *T*_p_ reached a plateau and showed no further increase (Figures 2c and S1). The open circles plotted in Figure 2c indicate the melting temperature (*T*_m_) of SE‐B, calculated numerically using the nearest‐neighbor model [24, 45] (see also Figures 1 and S2), where the *T*_m_ of SE‐B is defined as the temperature at which half of the SE‐B sequences dissociate. In the range of 0–12 nt, where *T*_p_ increased with SE‐B extension, the SE‐B *T*_m_ was lower than *T*_p_ (Figure 2c). Here, a single SE‐B binding is too unstable to connect S‐motifs, and the S‐motif connection is achieved by four SE‐A_4_ and two SE‐B_*j*_ of S‐motifs. In the range of 16–20 nt, where the plateau was observed, the SE‐B *T*_m_ exceeded *T*_p_ (Figure 2c), indicating that more than half of SE‐B_*j*_ hybridized and that S‐motif connectivity was nearly saturated. As a result, *T*_p_ becomes unresponsive to further extensions of SE‐B.

**FIGURE 3:**
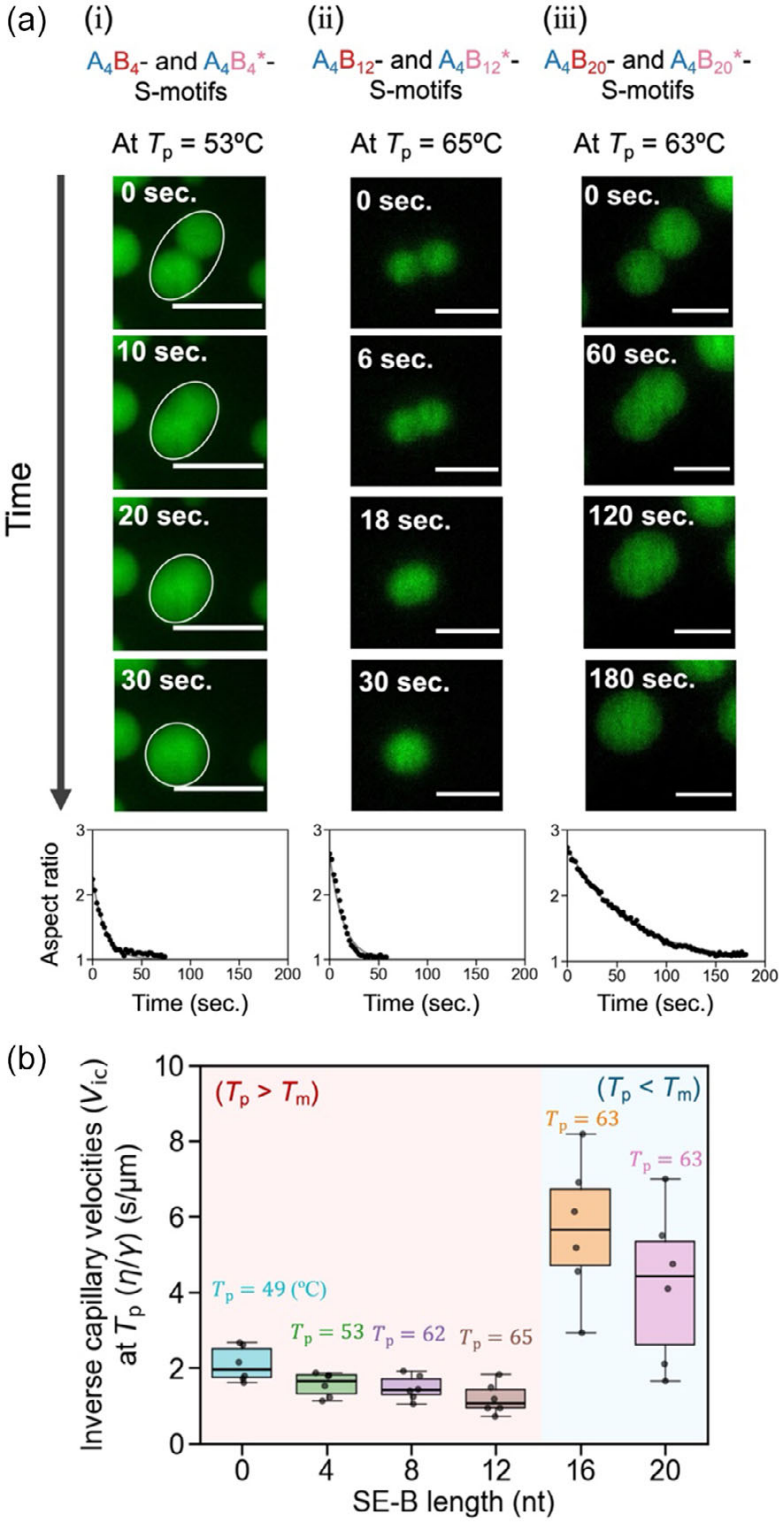
Fusion dynamics of DNA droplets at each phase‐separation temperature, *T*_p_. (a) Representative time‐lapse images consisting of (i) A_4_B_4_‐ and A_4_B_4_^*^‐S‐motifs, (ii) A_4_B_12_‐ and A_4_B_12_^*^‐S‐motifs, and (iii) A_4_B_20_‐ and A_4_B_20_^*^‐S‐motifs. The white ellipse indicates the fitted ellipse for the fusing droplet. Scale bars: 20 μm. (b) Box plot of SE‐B sequence‐dependence of *V*_ic_ at each *T*_p_ (n=6). *V*_ic_ was analyzed from (a, bottom) the time‐dependent change in the aspect ratio of fusing droplets.

Next, to explore how the dynamic properties depend on SE‐B length, the fusion process of DNA droplets at the phase‐separation temperature, *T*_p_, was observed (Figure 3). The fusion of the ten‐micrometer‐sized droplets composed of the A_4_B_4_‐ and A_4_B_4_*‐S‐motifs (Figure 3a‐i) and those composed of the A_4_B_12_‐ and A_4_B_12_*‐S‐motifs (Figure 3a‐ii) was completed in approximately 30 s. In contrast, droplets composed of the A_4_B_20_‐ and A_4_B_20_*‐S‐motifs took approximately 180 s to fuse (Figure 3a‐iii). To analyze the fusion process, the fusing droplets were fitted with an ellipse at each time point (white ellipses in Figure 3a‐i), and the aspect ratio of the ellipse was tracked over time, as previously reported [29, 46, 47]. An exponential curve (Equation S1 in the Supporting Information) served as the fitting function for the changes in aspect ratios over time (Figure 3a, bottom). The inverse capillary velocity (*V*_ic_ =η/γ), defined as the ratio between the droplet viscosity (η) and surface tension (γ), was estimated through curve fitting (Section 1.5 in the Supporting Information) [48].

Interestingly, within the 0–12 nt range of SE‐B, *V*_ic_ showed no statistically significant change despite the increase in *T*_p_ (Figures 3b and S3; Table S3). At 16 and 20 nt, where *T*_p_ exceeded the SE‐B *T*_m_, *V*_ic_ showed much larger values than those at 0–12 nt (Figures 3b and S3), likely due to the higher viscosity (η) from more stable SE‐B binding. These results suggest that stabilizing SE‐B interactions increased thermal stability without affecting fusion dynamics when *T*_p_ was higher than the SE‐B *T*_m_ (SE‐B length: 0–12 nt), whereas stabilizing SE‐B interactions increased thermal stability and slowed the fusion dynamics (SE‐B length: 16–20 nt).

**FIGURE 4:**
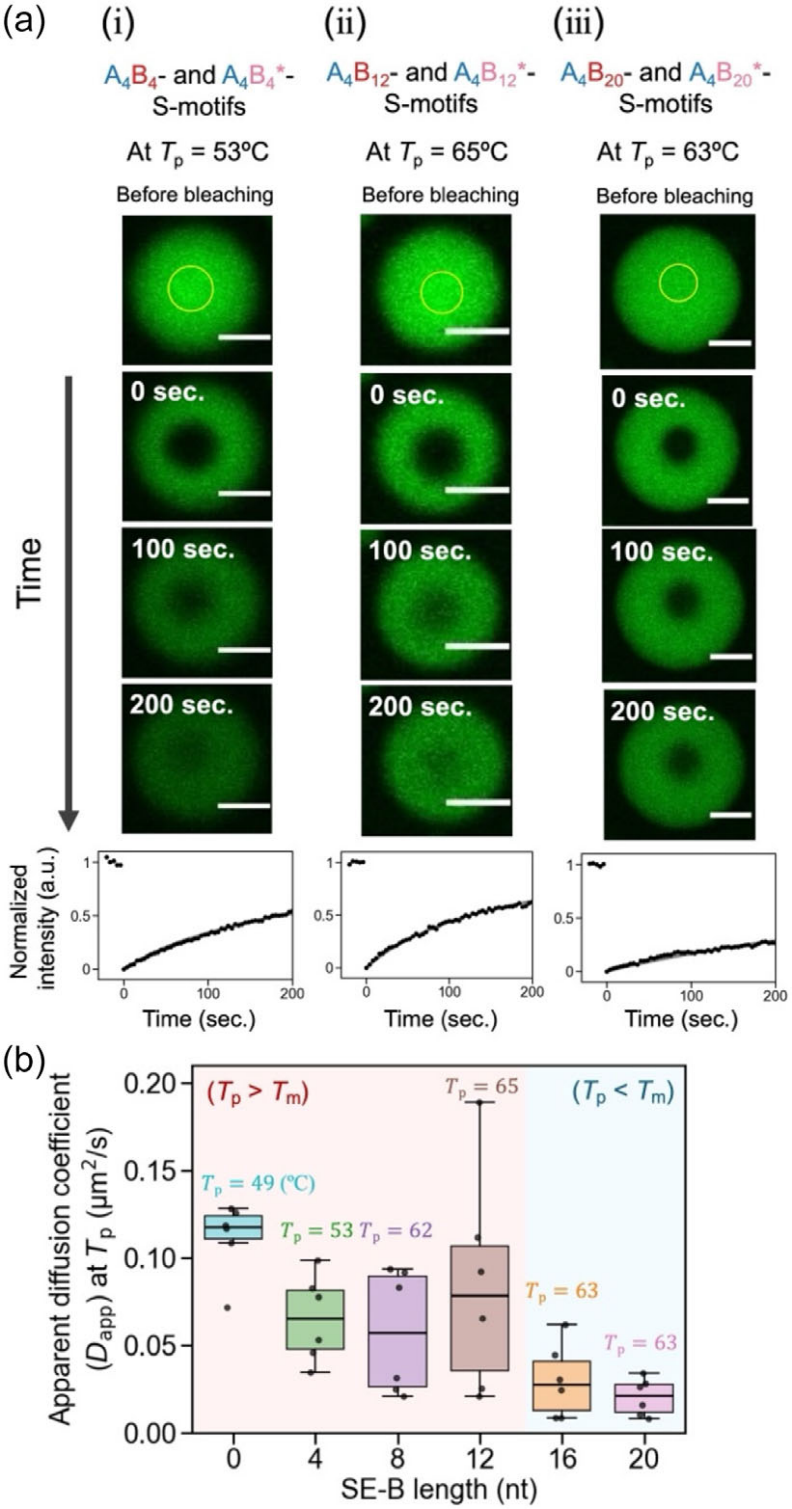
Internal mobility of S‐motifs within a DNA droplet at each phase‐separation temperature, *T*_p_. (a) Representative images of the FRAP experiments for a droplet composed of (i) A_4_B_4_‐ and A_4_B_4_^*^‐S‐motifs, (ii) A_4_B_12_‐ and A_4_B_12_^*^‐S‐motifs, and (iii) A_4_B_20_‐ and A_4_B_20_^*^‐S‐motifs**.** The yellow circles indicate bleached areas. Scale bars: 10 μm. (b) Box plot of SE‐B length‐dependent *D*_app_ at each *T*_p_ (n=6). *D*_app_ was analyzed using time‐dependent fluorescence recovery after photobleaching (a, bottom).

To explore another dynamic property of DNA droplets, the internal mobility of S‐motifs was measured through fluorescence recovery after photobleaching (FRAP) experiments at each *T*_p_ (Figure 4). Following the bleaching of the center region of the DNA droplets (yellow circles in Figure 4a, top), the recovery of fluorescence intensity was measured. The photobleached regions, with a radius of ∼ 5 µm, recovered to approximately half of the initial intensity within 200 s for droplets composed of A_4_B_4_‐ and A_4_B_4_*‐S‐motifs (Figure 4a‐i) and those composed of A_4_B_12_‐ and A_4_B_12_*‐S‐motifs (Figure 4a‐ii). In contrast, the photobleached regions of DNA droplets composed of A_4_B_20_‐ and A_4_B_20_*‐S‐motifs showed minimal recovery (Figure 4a‐iii). The apparent diffusion coefficient (*D*_app_) of the S‐motifs was derived from the recovery curves (Figures 4a (bottom) and S4). For A_4_B_*j*_‐ and A_4_B_j_*‐S‐motifs (*j* = 0, 4, 8, and 12), no significant difference in *D*_app_ was observed (Figures 4b and S4; Table S4). For *j* = 16 and 20, relatively lower *D*_app_ values were observed (Figures 4b and S4; Table S4), corroborating the slower fusion dynamics in these droplets (Figure 3b). This would be mainly because the SE‐B binding remains stable when the observation temperature (≈ *T*_p_) was below *T*_m_.

Collectively, these results suggest that DNA droplet stabilization around *T*_p_ may rely on different mechanisms depending on the SE‐B *T*_m_: (i) In the range of *j* = 0–12 (*T*_p_ > *T*_m_), S‐motifs undergo self‐assembly to form droplets despite the SE‐B *T*_m_ being lower than *T*_p_, suggesting the involvement of cooperative yet unstable multivalent interactions among all SEs, including four SE‐A_4_s and two SE‐B_*j*_s. According to the high internal mobility of S‐motifs observed by FRAP experiments (Figure 4), all the SEs are likely continually being attached and detached. (ii) In contrast, in the range of *j* = 16–20 (*T*_p_ < *T*_m_), where the SE‐B *T*_m_ exceeds *T*_p_, relatively stable interactions involving two SE‐B_*j*_s with weaker supports from four SE‐A_4_s are suggested. In this regime, FRAP analysis indicates relatively slow internal mobility (Figure 4), which is consistent with an interpretation in which SE‐A_4_ is continually being attached and detached, while SE‐B_*j*_ remains mostly attached.

The results shown in Figure 3–4 demonstrate that maintaining the length of SE‐A while extending SE‐B increased the thermal stability of the DNA droplets without significantly affecting their dynamic properties at *T*_p_, such as droplet fusion and internal mobility of the S‐motifs. It was also revealed that when the length of SE‐B exceeds the length at which *T*_m_ exceeds *T*_p_, the dynamic properties of the droplet are inhibited. The notable transition in the dynamic properties observed between 12 and 16 nt for SE‐B could be attributed to a sigmoidal increase in SE hybridization as the sequence length increased. This phenomenon can be interpreted as an approximately two‐state response, governed by the relative relationship between *T*_m_ and *T*_p_. In addition, this is expected to be primarily attributed to the polymerization (i.e., chain formation) of the S‐motif owing to stable SE‐B binding. When S‐motif chains are formed, the molecules tend to become entangled, restricting the movement of the S‐motif within the droplet. To evaluate the degree of polymerization, agarose gel electrophoresis was conducted on the A_0_B_*j*_‐ and A_0_B_j_*‐S‐motifs (*j* = 4, 8, 12, 16, and 20) (Note that SE‐A_0_ was used for four arms to prevent condensation) (Figure S5), resulting in the observation of S‐motif multimers. However, it is important to note that the electrophoresis was performed at a room temperature, which is lower than *T*_p_.

To examine the binding behavior of the S‐motif, a coarse‐grained Brownian dynamics simulation [49, 50] was also conducted (Figure S6). The numerical model successfully reproduces the dependence of the *T*_p_ trend on the SE‐B length (Figures S7 and S8). Analyzing the number of S‐motifs bound by SE‐B at the phase‐separation temperature in simulation revealed that the average degree of polymerization of S‐motifs increased as SE‐B lengthened (Figures S9 and S10).

## 3 Conclusion

In summary, we demonstrated that the thermal stability can be controlled semi‐independently of the liquid‐like dynamics in DNA droplets by selectively extending only two of the six SEs on S‐motifs. Increasing the SE‐B length raised *T*_p_ without affecting either droplet fusion or internal mobility of the S‐motif at *T*_p_, when the SE‐B *T*_m_ remained below *T*_p_ (4–12 nt). This approach is useful when one aims to maximize fusion dynamics and internal mobility within condensates at the highest possible temperature. In contrast, the fusion dynamics slowed and internal mobility decreased when the SE‐B *T*_m_ exceeded *T*_p_ (16–20 nt). Gel electrophoresis and simulations suggest that SE‐B stabilization induces chain‐like polymerization of the S‐motif, likely hindering its dynamic behaviors.

Semi‐independent programmability refers to the ability to modify the thermal stability (*T*_p_) of DNA droplets without affecting their dynamic properties at *T*_p_. Since liquid‐like dynamic properties—such as droplet fusion, division, and molecular exchange—are crucial for the function of condensates, it is advantageous to maintain these dynamic properties while adjusting *T*_p_. This would be particularly beneficial in environments where droplet formation is hindered by environmental or buffer conditions. Additionally, the observation of reduced dynamic properties under similar *T*_p_ conditions (8‐20nt) for longer SE‐B (>16 nt) indicates potential applications as a stimulus–responsive material. This responsiveness can be achieved by integrating light‐responsive molecules, DNA strand displacement reactions, and ligase/nuclease‐mediated reactions to reconfigure the SE.

The sharp transition observed in dynamics might also be linked to variations in the polymerization degree of the S‐motif. However, achieving precise control over the polymerization degree in this system remains elusive, and we view this as a significant future challenge for more rigorously programming dynamic changes. Furthermore, although our analysis assumed that the contributions of SE‐A and SE‐B were additive, it is crucial to recognize the potential for nonlinear effects in multivalent interaction systems. The impact of design modifications in SE‐B on the contribution of SE‐A remains a subject for future investigation. It would be also beneficial to consider other lengths of SE‐A. In addition, while this study employed *T*_m_ as a thermodynamic parameter, further investigation into sequence dependence is necessary and useful.

The capability to program DNA droplets with both stable and dynamic properties through DNA sequence design is highly beneficial in the field of bottom‐up synthetic biology, facilitating applications in artificial cells, molecular robotics, and microsized soft robotics. We believe that the concept of partial modification of multivalent monomers consisting biomolecular condensates will be extended to not only DNA condensates but also peptide‐, protein‐, or synthetic polymer‐based condensates.

## Supporting Information

Additional supporting information can be found online in the Supporting Information section.

## Funding

This work was supported by MEXT/JSPS KAKENHI (Nos. JP24H00070 and JP25H01361 to M.T.), Human Frontier Science Program (HFSP; RGP0016/2022‐102 to M.T.), and Research Fellowships of JSPS for Young Scientists (No. JP24KJ1076 to N.Y.) and partially supported by JST Adopting Sustainable Partnerships for Innovative Research Ecosystem (ASPIRE) (No. JPMJAP24B4 to M.T.).

## Conflicts of Interest

The authors declare no conflicts of interest.

## Supporting information

Supplementary Material

## Data Availability

The data that support the findings of this study are available in the supplementary material of this article.
