# Supplementary material for: Semi‐Independent Control of Stability and Mobility in DNA Condensates
Source: Chembiochem. 2026 Feb 23;27(4):e202500927. doi: 10.1002/cbic.202500927 (PMC12928539; doi:10.1002/cbic.202500927)
Supplement: Supplementary file 1 — Supplementary Material [file CBIC-27-e202500927-s001.pdf]

Supporting Information

Semi-independent Control of Stability and Mobility in DNA Condensates

Naoki Yoshida[1], Kei Goraku[2], Ryohei Furuichi[2], Mitsunori Takano[3], Yusuke Sato[4], Masahiro Takinoue[1,2,5,6,*]

[1]Department of Life Science and Technology, Institute of Science Tokyo, 4259 Nagatsuta-cho, Midori-ku, Yokohama, Kanagawa 226-8501, Japan

[2]Department of Computer Science, Institute of Science Tokyo, 4259 Nagatsuta-cho, Midori-ku, Yokohama, Kanagawa 226-8501, Japan

[3]Department of Pure and Applied Physics, Waseda University, 3-4-1 Ohkubo, Shinjuku-Ku, Tokyo 169-8555, Japan

[4]Department of Intelligent and Control Systems, Kyushu Institute of Technology, 680-4 Kawazu, Iizuka, Fukuoka, 820-8502, Japan

[5]Research Center for Autonomous Systems Materialogy (ASMat), Institute of Integrated Research, Institute of Science Tokyo, 4259 Nagatsuta-cho, Midori-ku, Yokohama, Kanagawa 226-8501, Japan

[6]Laboratory for Chemistry and Life Science, Institute of Integrated Research, Institute of Science Tokyo, 4259 Nagatsuta-cho, Midori-ku, Yokohama, Kanagawa 226-8501, Japan

*Corresponding author: Laboratory for Chemistry and Life Science, Institute of Integrated Research, Institute of Science Tokyo, 4259 Nagatsuta-cho, Midori-ku, Yokohama, Kanagawa 226-8501. Email: takinoue@cls.iir.isct.ac.jp

1. Experimental Section

1.1 Material

Sodium chloride, bromophenol blue (BPB; 029-02912), Xylene Cyanol FF (XC; 244-00461), glycerol (075-00616), and bovine serum albumin (BSA; 019-15123) were purchased from FUJIFILM Wako Pure Chemical Corp. (Osaka, Japan). Tris-HCl (15568025) and SYBR Gold Nucleic Acid Gel Stain solution (S11494) were obtained from Thermo Fisher Scientific (Tokyo, Japan). Mineral oil (23334-85) and EDTA (35430-61) were purchased from Nacalai Tesque, Inc. (Kyoto, Japan). TBE (318-90041) was purchased from Nippon Gene Co., Ltd. (Tokyo, Japan). A 2% agarose gel (AG42TB) was purchased from Funakoshi Co., Ltd. (Tokyo, Japan). 100 bp and 1 kb DNA ladders (N0467S and N0552S, respectively) were obtained from New England BioLabs Inc. (MA, USA). The DNA sequences listed in Tables S1-2 were purchased from Eurofins Genomics (Tokyo, Japan). The fluorescently labeled DNA was of high-performance liquid chromatography (HPLC) grade, while the others were of oligonucleotide purification cartridge (OPC) grade. The 2× loading buffer (0.05% BPB, 0.05% XC, 15% glycerol, and 20 mM EDTA (pH 8.0)) was prepared using the above-mentioned reagents. Ultrapure water was generated using Direct-Q UV from Merck Millipore Corp. (MA, USA). Glass slides (dimensions: 30 × 40 mm; thickness: 0.17 mm; C030401) and coverslips (dimensions: 18 × 18 mm; thickness: 0.17 mm; C218181) were obtained from Matsunami Glass (Kishiwada, Japan). Double-sided tapes with thicknesses of 30 μm (707#4) and 1 mm (T-4613) were purchased from Teraoka Seisakusho Co., Ltd. (Tokyo, Japan) and Nitoms, Inc. (Tokyo, Japan), respectively.

1.2 DNA nanostructure design

The DNA sequences were designed using the Nucleic Acid Package (NUPACK)[1]. We designed two types of six-branched motifs (S-motifs), A_4B_j- and $A_4B_j^*$-S-motifs, each assembled from six single-stranded DNAs. An S-motif can self-assemble into macromolecular structures via SE interactions[2]. Self-complementary SE-A on the top and bottom four branches was designed with 4 nt. Six types of SE-B and their complementary SE-B*, on the left and right branches, respectively, were designed with 0, 4, 8, 12, 16, or 20 nucleotides. Note that SE-A was configured to avoid crosstalk with SE-B and SE-B*. Two S-motifs were constructed to prevent intramolecular hybridization between SE-B and SE-B* within an S-motif.

1.3 Sample preparation for microscopic observation

Unless explicitly indicated, the experiments were performed using a buffer containing 20 mM Tris-HCl and 350 mM NaCl (pH 7.5). DNA strands were mixed in a test tube at 2.5 μM each in the buffer solution. Notably, fluorescently labeled (6-carboxyfluorescein, 6-FAM) DNA was included at a 20% molar concentration instead of non-labeled DNA; that is, (α-Six-5_4): (α-Six-5_0_FAM) = 80:20 (Figure S1a).

1.4 Measurement of the phase-separation temperatures (*T_p*)

Observation chambers were prepared as described in previous studies[2,3]. The glasses and coverslips were soaked in 5% (w/v) BSA dissolved in 20 mM Tris-HCl (pH 7.5) for over 30 min. After coating with BSA, the glasses and coverslips were washed with ultrapure water and dried. The BSA-coated glasses and coverslips were assembled using double-sided tape (30 μm thick). The sample solution, prepared as described in Section 1.3, was loaded into the space between the coated glass and coverslips. The edges of the coverslips were coated with a manicure paste. To prevent evaporation during observation, the chamber was covered with mineral oil using a double-sided tape bank (1 mm thick). The samples in the observation chamber were visualized using a fluorescent microscope (IX-71, Olympus, Tokyo, Japan) equipped with a spinning-disk confocal system (CSU-X1, Yokogawa, Tokyo, Japan), an electron-multiplying charge-coupled device (EM CCD) camera (iXon X3, Andor), and a stage heater (10021-PE120 system, Linkam, Fukuoka, Japan). Samples containing 6-FAM were observed using an excitation wavelength of 473 nm.

The sample solution loaded into the chamber was incubated at 85°C for 3 min using the stage heater. The temperature was then lowered at a rate of 1°C/min, and the sample was observed at every 1°C interval. To determine T_p, the number of droplets was counted from each captured image using ImageJ's "Analyze Particles" (AP) method. The images were binarized using a 99th percentile threshold to eliminate background signals. Droplets with an area (referred to as "Size" in AP) of at least 1 μm^2 were counted, and the temperature at which the number of droplets reached five or more was defined as T_p to avoid miscounting caused by image noise.

1.5 Observation of fusion events and analysis

BSA-coated glasses were prepared following the procedure described in Section 1.4. Double-sided tape (thickness: 1 mm) with 5 mm-diameter holes was placed on the BSA-coated glass. The sample prepared in Section 1.3 was loaded into the hole of the observation chamber and covered with mineral oil to prevent evaporation. The same visualization equipment as described in Section 1.4 was used. The chamber containing the sample was incubated at 85°C for 3 min and then cooled below each T_p at a rate of –1°C/min. After preincubation for 5 min at each T_p, the fusion events of the DNA droplets were observed at a scanning velocity of 2 s per frame and analyzed using AP. Sequential images were binarized using the 85th percentile threshold to eliminate the background intensity. The droplets undergoing fusion were approximated as ellipses via "Fit Ellipse" in AP. The aspect ratio ("AR" in AP), which is the ratio of the long to short axes, was then calculated. The time evolution of the aspect ratio was fitted using the following exponential curve[4,5]:

$$f(t) = 1 + (A_0 - 1) \cdot \exp(-t/\tau_{\text{fusion}}), \quad \text{(S1)}$$

where $f(t)$ is the aspect ratio at time t, A_0 is the initial aspect ratio, and τ_{fusion} is the characteristic time.

V_{ic}, which is the ratio of the viscosity to the surface tension of the droplets, was estimated from the relationship[6]:

$$\tau_{\text{fusion}} \approx (\eta/\gamma) \cdot l, \quad \text{(S2)}$$

where η is the viscosity, γ is the surface tension, and l is defined as

$[(l_{\text{long axis } (t=0)} - l_{\text{short axis } (t=0)}) \times l_{\text{short axis } (t=0)}]^{1/2}$ ($t = 0$: initiation of fusion).

1.6 Diffusion coefficient measurements

As described in Section 1.5, observation chambers containing the samples were constructed. Samples were visualized using a confocal laser scanning microscope (CLSM) (FV-1000, Olympus, Tokyo, Japan) and the same stage heater as described in Section 1.4.

The diffusion coefficients of the S-motifs in the DNA droplets were measured using FRAP experiments[2,3]. The chamber containing the sample was placed on the stage heater. The temperature was maintained at 85°C for 3 min and then decreased at a rate of –1°C/min to below each T_p. FRAP experiments were conducted at each T_p after a 5-minute preincubation. Visualization was performed 4-second interval between images. Five images were captured before photobleaching a circular region of interest (ROI) centered on the droplet. Subsequently, fluorescence recovery in the ROI and non-bleached reference region within the droplets was monitored over time and analyzed using ImageJ. The fluorescence intensity of the reference

region was used to normalize the intensity of ROI[4,5]. The resulting normalized data were fitted to the following equation[4,5]:

$$I(t) = \frac{\alpha+\beta\left(\frac{t}{\tau_{1/2}}\right)}{1+\left(\frac{t}{\tau_{1/2}}\right)}, \tag{S3}$$

where *I*(*t*) is the intensity at time *t*, $\tau_{1/2}$ is the recovery half-time constant, and α and β are fitting parameters. The apparent diffusion coefficient (D_{app}) was calculated using the following equation[4,5]:

$$D_{\mathrm{app}} = r^2/\tau_{1/2}, \tag{S4}$$

where *r* is the radius of ROI.

1.7 Gel electrophoresis and motif polymerization analysis

Samples were prepared in a buffer containing 20 mM Tris-HCl (pH 7.5) and 350 mM NaCl with 2.5 μM DNA strands. The sample was heated to 85°C for 3 min and then cooled to 25°C at a rate of –1°C/min using a thermal cycler (Mastercycler® nexus X2, Eppendorf, Germany). The samples were then diluted and mixed with 2× loading buffer at a 1:1 ratio. Electrophoresis samples were run on 2% agarose gels in 1× TBE buffer containing 5 mM $MgCl_2$ at 70 V for 70 min. After staining with a 1× SYBR Gold solution, the gels were imaged using a gel imager (FLA-5100, FUJIFILM Corporation, Tokyo, Japan).

1.8 Single-particle coarse-grained simulation

We developed a single-particle coarse-grained model that represents the S-motif as a single particle (Figure S6). Here, the Brownian dynamics model[7,8] was used to describe the particle motion driven by inter-particle interactions and thermal fluctuations. The following overdamped Langevin equation describes the motion of each particle:

$$\gamma\frac{d\vec{x}}{dt} = -\vec{\nabla}U(\vec{x}) + \vec{R}(t), \tag{S5}$$

where $\vec{x}$ denotes the position of a particle, t is time, γ is the friction coefficient, $\vec{\nabla}U$ is the inter-particle force, and $\vec{R}(t)$ denotes the stochastic force from the surrounding medium of the particle.

For the numerical simulations, this equation was discretized using the Euler–Maruyama method[9] as follows:

$$\vec{x}_{t+1} = \vec{x}_t - \frac{1}{\gamma}\vec{\nabla}U(\vec{x}_t)\Delta t + \vec{\xi}\sqrt{\frac{2k_{\mathrm{B}}T}{\gamma}\Delta t}, \tag{S6}$$

where k_B is the Boltzmann constant, T is the absolute temperature, and $\vec{\xi}$ follows a normal distribution with zero mean and unit standard deviation in each dimension. The harmonic potential $U(\vec{x}_t)$ is defined between the particles with respect to the inter-particle distance r (Figure S6). The simulations were performed with 700 particles at a concentration of 5 μM, and the simulation time step is $\Delta t = 1 \times 10^{-9}$ s.

The number of particles continuously connected through SE-B binding (number-average degree of particle polymerization, M_n) was calculated using the following equation:

$$M_n = \frac{\sum_{i=1}^{n_{max}} i \cdot N_i}{\sum_{i=1}^{n_{max}} N_i}, \tag{S7}$$

where n_{max} is the maximum degree of polymerization, and N_i is the number of polymers with polymerization degree i.

1.9 Statistical Analysis

Statistical analysis was performed to assess the inverse capillary velocity (V_{ic}) and the apparent diffusion coefficient (D_{app}). The values obtained from the methodologies detailed in Sections 1.6 and 1.7 were used without any prior data preprocessing. The data were displayed as box plots. The sample size for each condition was $n = 6$. A one-tailed Welch's t-test was applied for the analysis. Under the null hypothesis that stabilizing SE-B results in a more stable S-motif network, thereby reducing its dynamic properties, one-tailed statistical tests were employed. The p-values are provided in Tables S3 and S4. All statistical analyses were conducted using Python. In other experiments, measurements were conducted with $n = 3$, and standard errors are represented as error bars.

2. Supplementary Figures

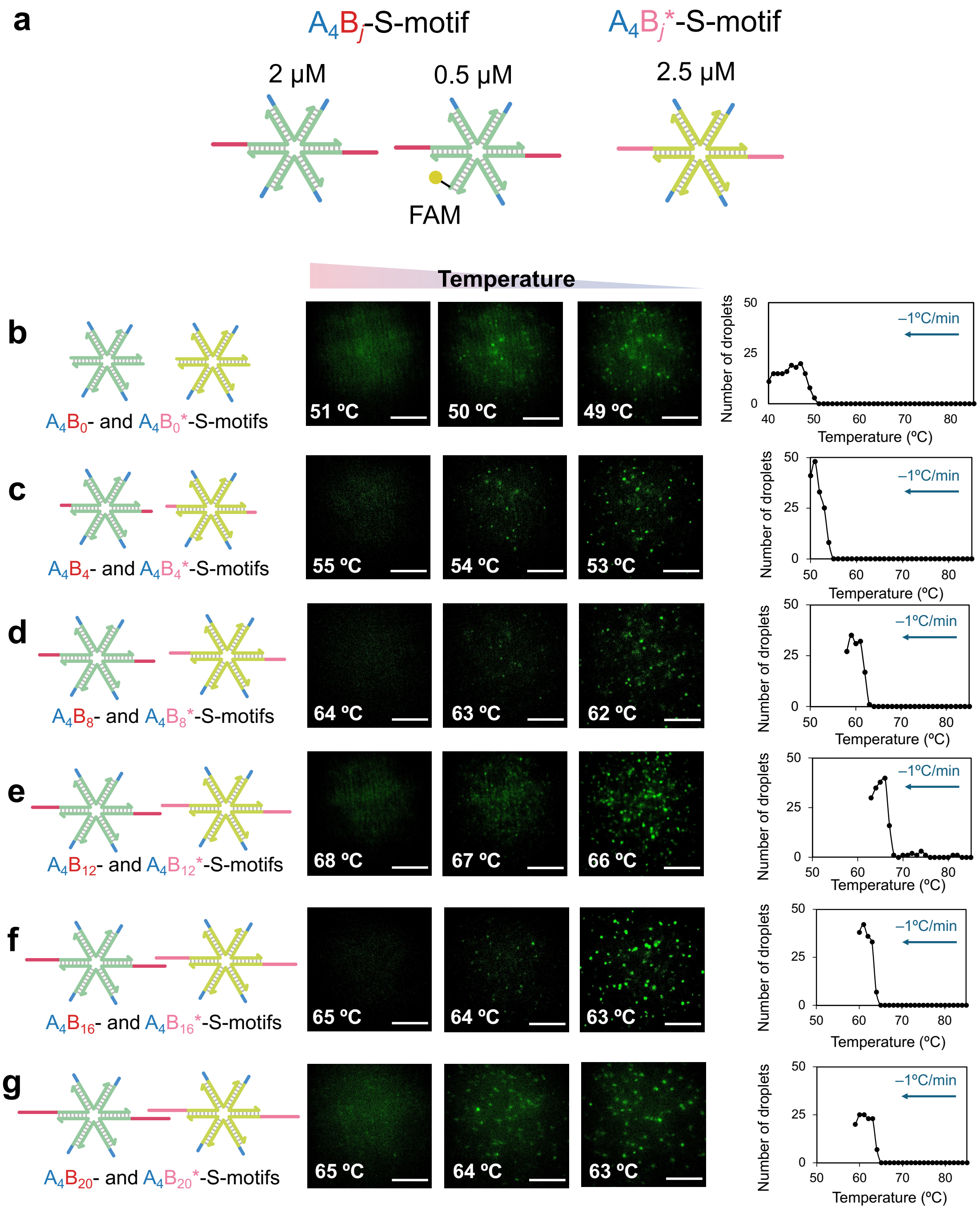

Figure S1. Sequential images of DNA droplet formation as the temperature decreased. (a) Fluorophore (FAM) labeling site on the S-motifs for visualization. (b) A_4B_0- and $A_4B_0^*$-S-motifs, (c) A_4B_4- and $A_4B_4^*$-S-motifs, (d) A_4B_8- and $A_4B_8^*$-S-motifs, (e) A_4B_{12}- and $A_4B_{12}^*$-S-motifs, (f) A_4B_{16}- and $A_4B_{16}^*$-S-motifs, and (g) A_4B_{20}- and $A_4B_{20}^*$-S-motifs. The adjacent plots illustrate the change in the number of counted droplets as the temperature decreased. Scale bars: 40 μm.

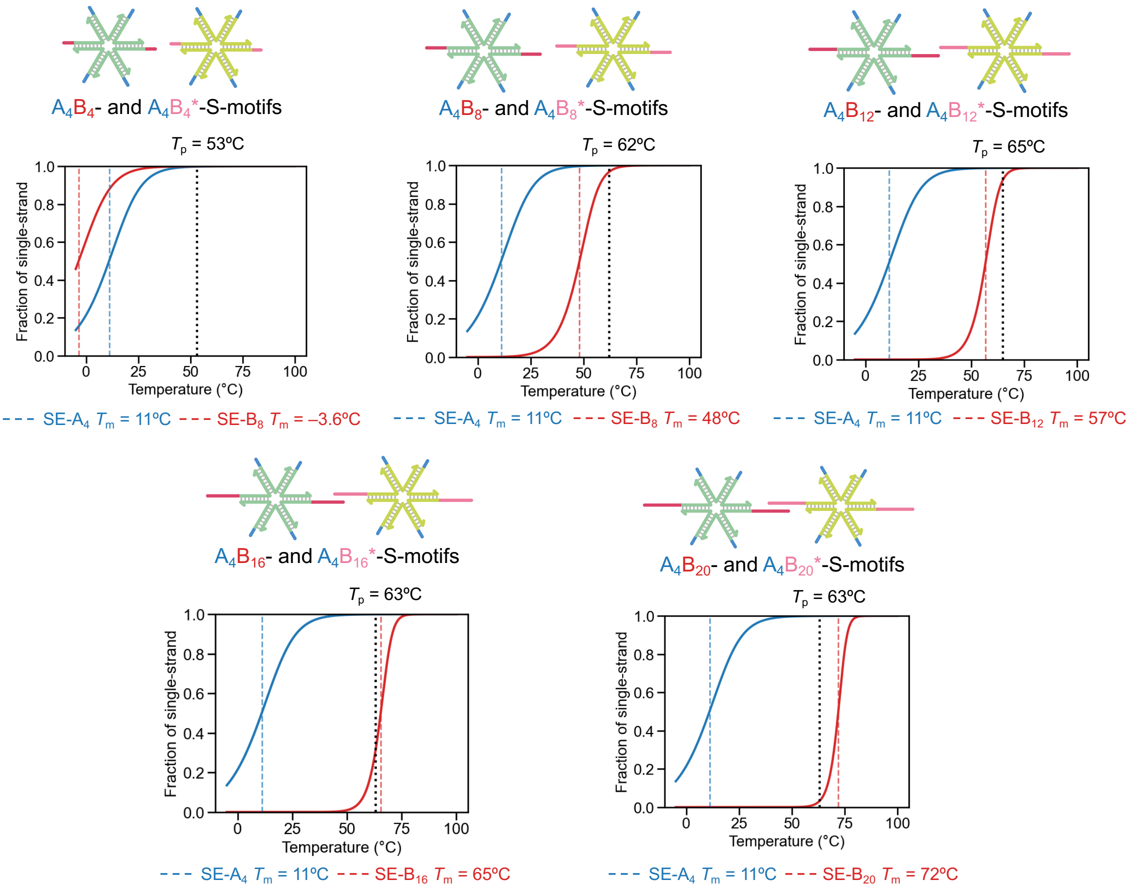

Figure S2. Melting curves and melting temperatures (T_m) for SE-A_4 and SE-A_4^* hybridization and SE-B_j and SE- B_j^* hybridization (j = 4, 8, 12, 16, and 20). The melting curves and T_m were calculated using the nearest-neighbor model and thermodynamic parameters for DNA[10] under the following condition: 10 μM SE-A, 5 μM SE-B, and 350 mM NaCl. The vertical black broken lines in each plot indicate the experimentally obtained T_p; the vertical blue and red broken lines indicate the calculated T_m for SE-A_4/SE-A_4^* and SE-B_j/SE- B_j^*, respectively.

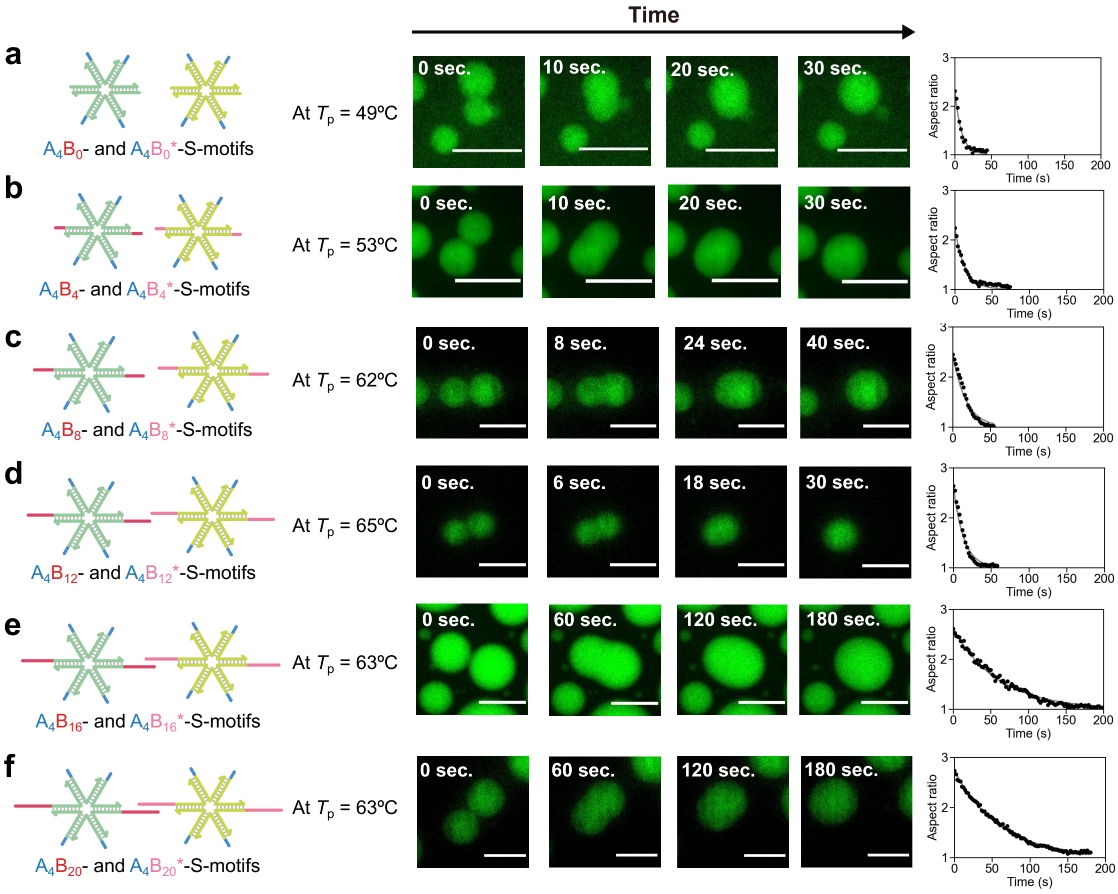

Figure S3. Representative sequential images of the fusion process (center) and aspect ratio change over time (right) for DNA droplets composed of (a) A_4B_0- and $A_4B_0^*$-S-motifs, (b) A_4B_4- and $A_4B_4^*$-S-motifs, (c) A_4B_8- and $A_4B_8^*$-S-motifs, (d) A_4B_{12}- and $A_4B_{12}^*$-S-motifs, (e) A_4B_{16}- and $A_4B_{16}^*$-S-motifs, and (f) A_4B_{20}- and $A_4B_{20}^*$-S-motifs. Scale bars: 20 µm. The experiments were conducted at each phase-separation temperature, T_p.

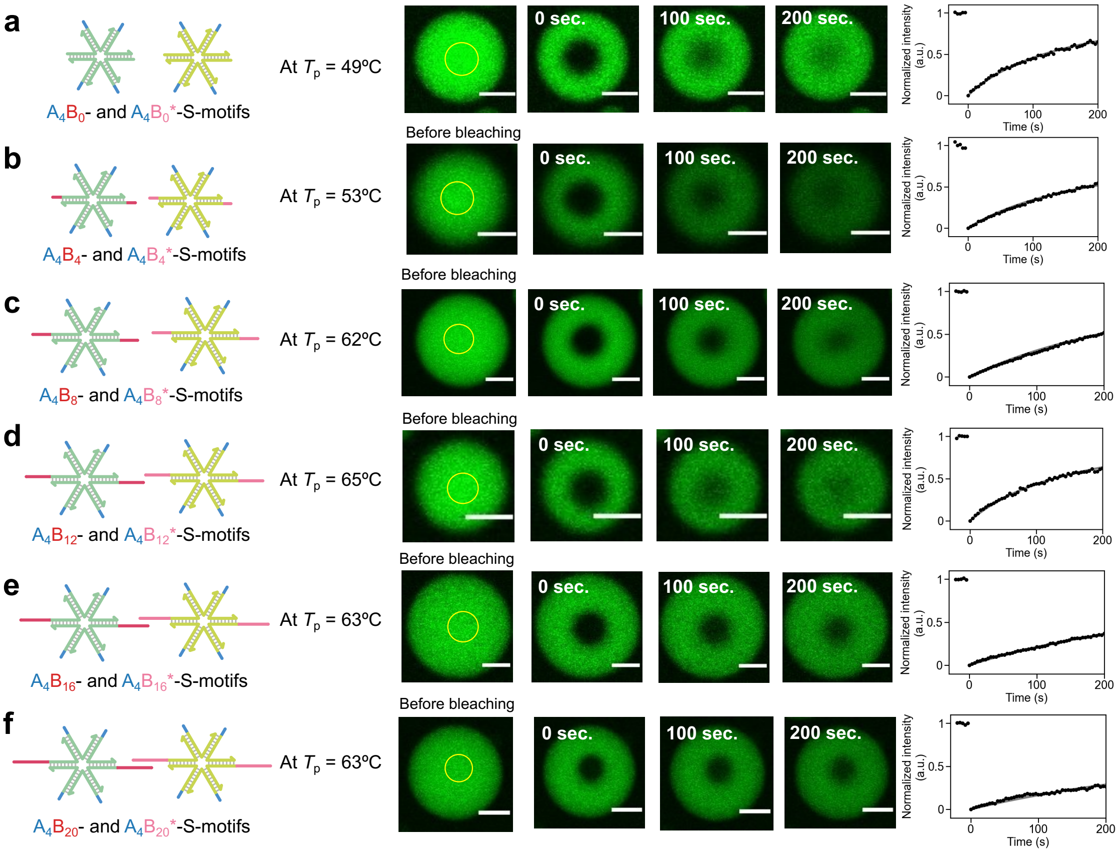

Figure S4. Fluorescence recovery after photobleaching results for the DNA droplets composed of (a) A_4B_0- and $A_4B_0^*$-S-motifs, (b) A_4B_4- and $A_4B_4^*$-S-motifs, (c) A_4B_8- and $A_4B_8^*$-S-motifs, (d) A_4B_{12}- and $A_4B_{12}^*$-S-motifs, (e) A_4B_{16}- and $A_4B_{16}^*$-S-motifs, and (f) A_4B_{20}- and $A_4B_{20}^*$-S-motifs. The yellow circles indicate the photobleached region. The right plots show the fluorescence intensity recovery curves for bleached regions. Scale bars: 10 µm. The experiment was performed at each phase-separation temperature, T_p.

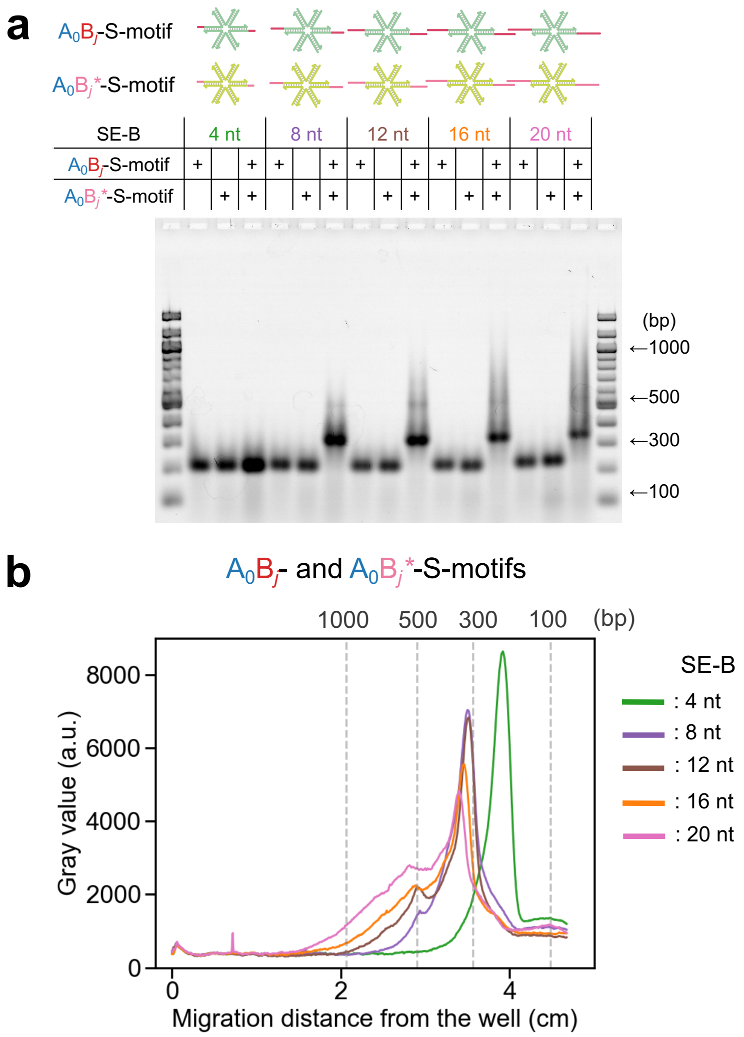

Figure S5. SE-B-mediated polymerization of the S-motif. (a) Agarose gel electrophoresis results and (b) intensity profiles derived from the gel images.

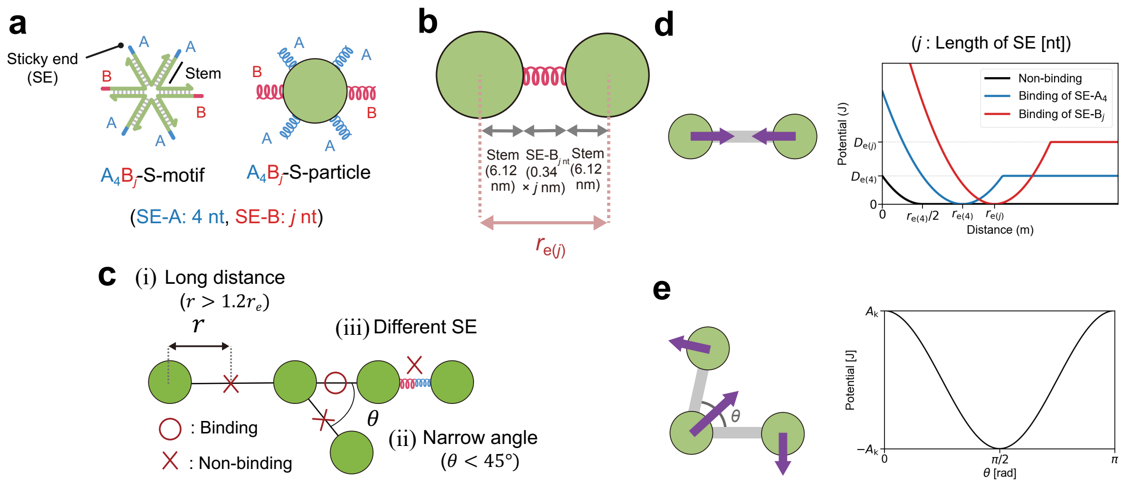

Figure S6. Single-particle coarse-grained simulation model. (a) The particle (green circle) represents a coarse-grained S-motif consisting of four SE-A_4 and two SE-B_j (i.e., A_4B_j-S-motifs) (j = 0, 4, 8, 12, 16, and 20). For simplicity, the coarse-grained S-motif particle represents both A_4B_j-S-motif and $A_4B_j^*$-S-motif in a non-distinguished manner. Thus, the particles directly interact with each other, and the interaction is based on a spring force following a potential $U(r)$. (b) The equilibrium bond distance ($r_{e(j)}$) depends on the stem length (18 nt, $0.34 \times 18 = 6.12$ nm) of the S-motif (a) and the sequence lengths of SE-A and SE-B. (c) Bonding conditions between particles. Bonds are not formed in the following three cases: (i) when the inter-particle distance exceeds 1.2 times the equilibrium bond distance, (ii) when the bond angle formed by three particles is 45° or less, and (iii) when the SE types are different. (d) The potential is composed of the distance-dependent potential ($U_r(r)$) and the angle-dependent potential ($U_\theta(\theta)$): $U(r) = U_r(r) + U_\theta(\theta)$. Distance-dependent potential between particles: $U_r(r) = k_H \cdot (r - r_{e(j)})^2$(when unbound, $r < r_{e(4)}/2$), 0 (when unbound, $r \geq r_{e(4)}/2$), $\frac{D_e}{(0.5r_{e(j)})^2} \cdot (r - r_{e(j)})^2$ (when bound, $r < 1.5r_{e(j)}$), D_e (when bound, $r \geq 1.5r_{e(j)}$). The D_e represents the depth of the well in (d) the harmonic potential. The correspondence between D_e and the SE length is shown in Table S5. (e) Angle-dependent potential for inter-particle interactions: $U_\theta(\theta) = k_A \sin 2\theta$. The parameters used in the simulations are listed in Table S5.

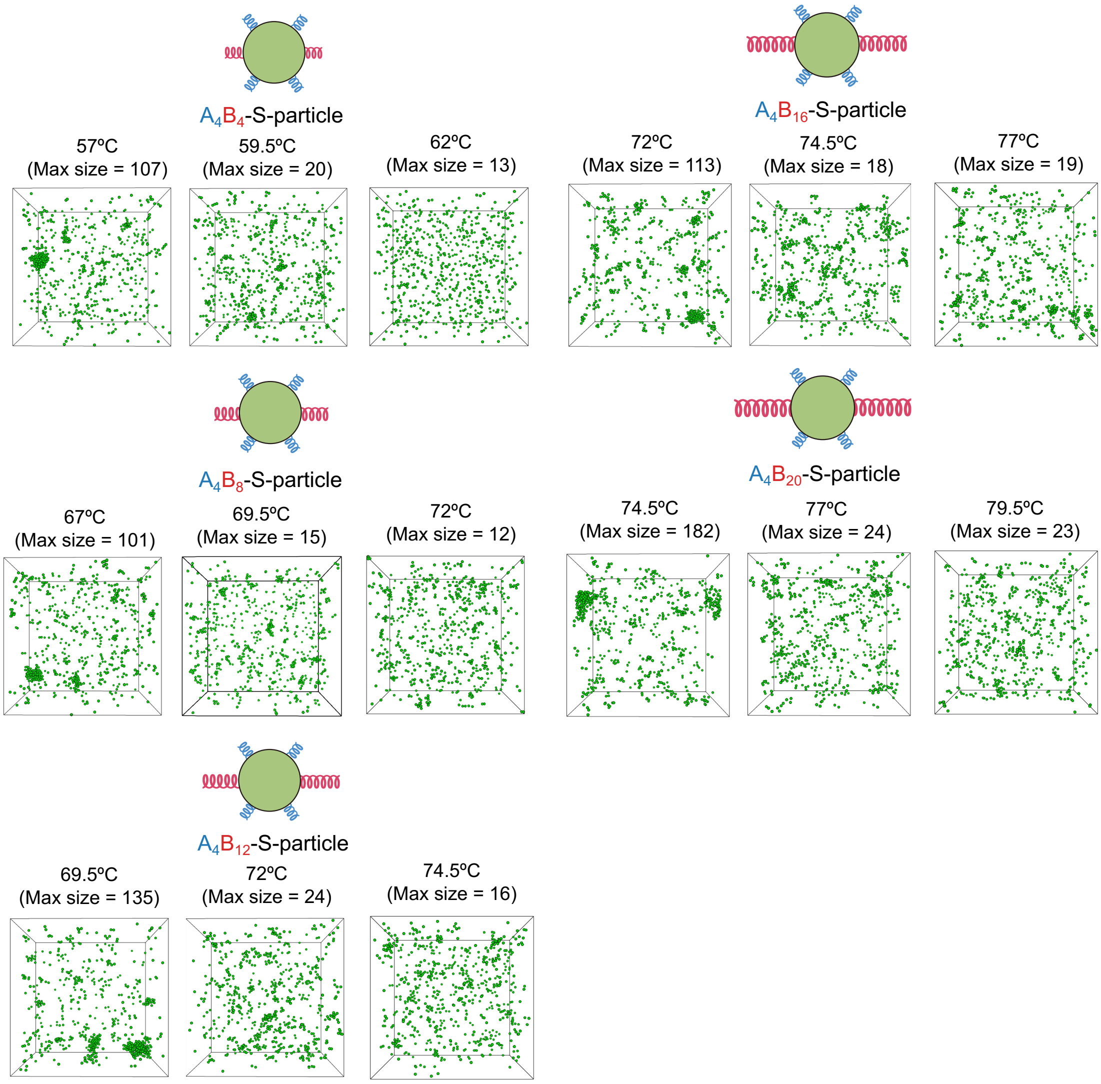

Figure S7. Temperature-dependent particle behavior in numerical simulations. Snapshots were captured 5 ms after the start of the simulations. ‘Max size’ indicates the number of particles in the largest-bonded condensates.

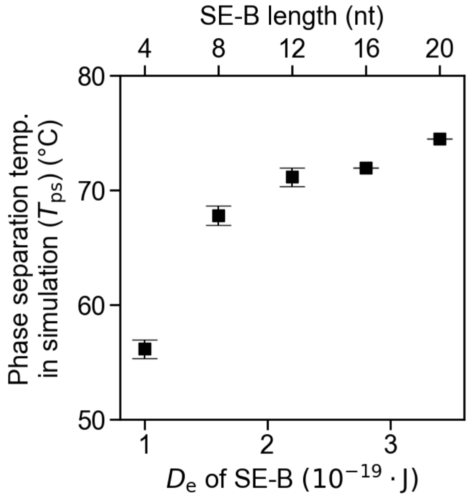

Figure S8. Phase-separation temperature in simulation (T_{ps}) was determined from the temperature-dependent behavior of A_4B_j- and $A_4B_j^*$-particles (Figure S7) as the temperature at which the largest condensate contained more than 50 particles. Error bars represent standard error ($n = 3$).

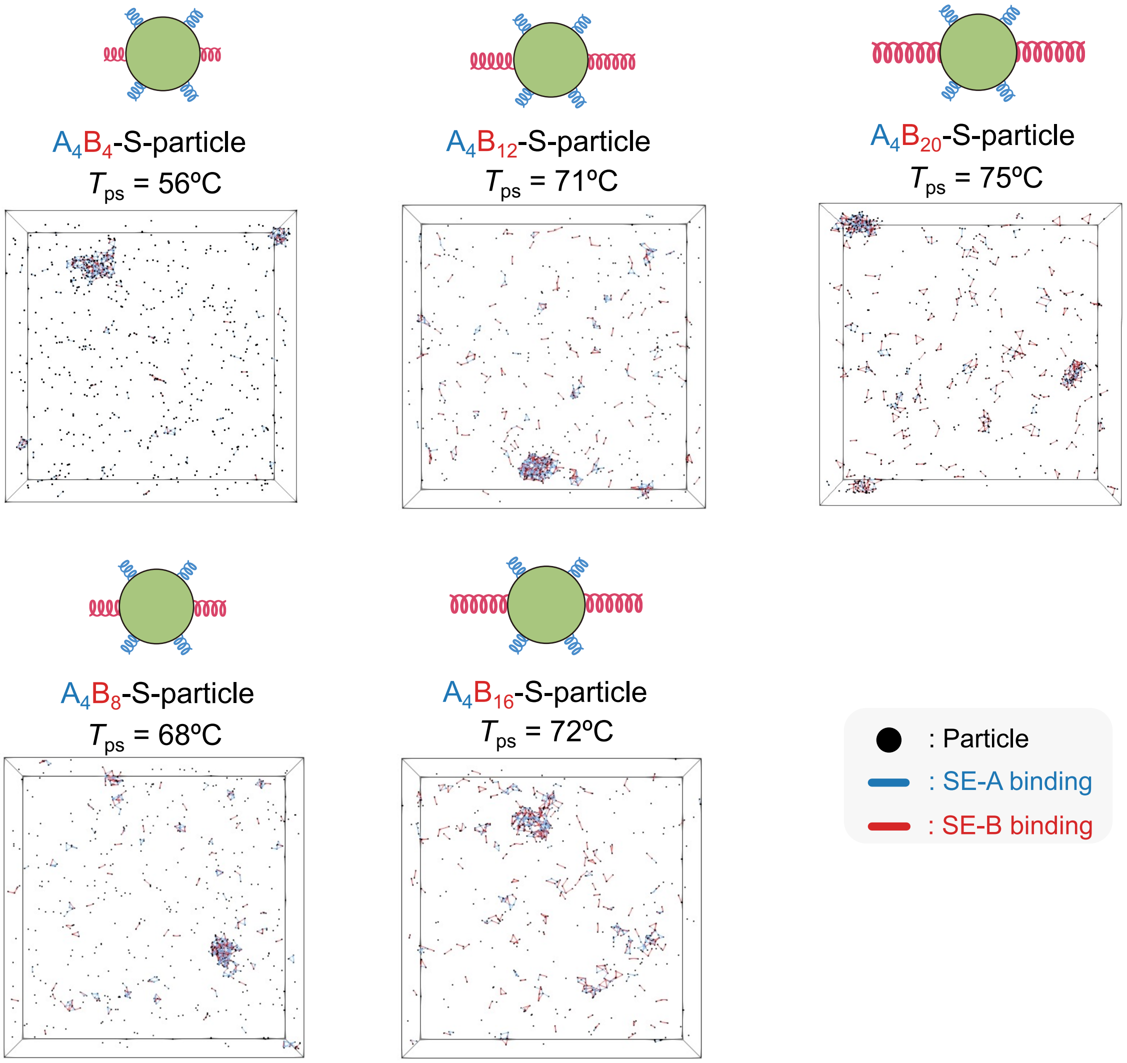

Figure S9. Simulation snapshot 5 ms after the start, showing the bonding state between particles. Simulations were performed at T_{ps} for each particle.

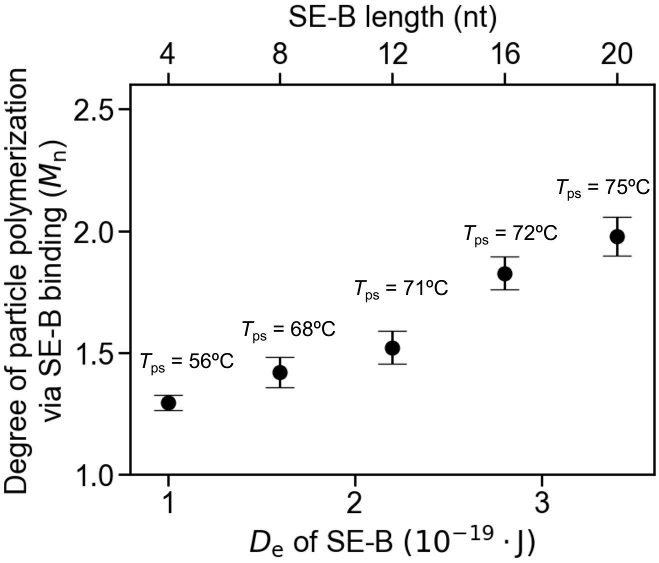

Figure S10. Number-average degree of particle polymerization via SE-B binding (M_n). Calculated at each particle T_{ps} when varying the SE-B length (Figure S9). Error bars indicate standard error ($n = 3$).

3. Supplementary Tables

Table S1. Oligonucleotide sequences of A_4B_j- and $A_4B_j^*$-S-motifs. In the tables below, A_4B_j-S-motifs are denoted as α, and $A_4B_j^*$-S-motifs as β.

**Name**	**Sequence (5'→ 3')**
α-Six-1_4	GCGCGCTGGACTAACGGAACGGTTAGTCAGGTATGCCAGCAC
α-Six-2_4	GCGCCTCAGAGAGGTGACAGCATTCCGTTCCGTTAGTCCAGC
α-Six-3_0	CCATGGTCCCAAGTGATGTTTGCTGTCACCTCTCTGAG
α-Six-3_4	CCGGCCATGGTCCCAAGTGATGTTTGCTGTCACCTCTCTGAG
α-Six-3_8	CCGGGCACCCATGGTCCCAAGTGATGTTTGCTGTCACCTCTCTG AG
α-Six-3_12	CCGGGCACTTATCCATGGTCCCAAGTGATGTTTGCTGTCACCTC TCTGAG
α-Six-3_16	CCGGGCACTTATAGCTCCATGGTCCCAAGTGATGTTTGCTGTCA CCTCTCTGAG
α-Six-3_20	CCGGGCACTTATAGCTGTCGCCATGGTCCCAAGTGATGTTTGCT GTCACCTCTCTGAG
α-Six-4_4	GCGCCGGCGCTGTAAATTTGCGTTCATCACTTGGGACCATGG
α-Six-5_4	GCGCCAGACGTCACTCTCCAACTTCGCAAATTTACAGCGCCG
α-Six-6_0	GTGCTGGCATACCTGACTTTGTTGGAGAGTGACGTCTG
α-Six-6_4	CCGGGTGCTGGCATACCTGACTTTGTTGGAGAGTGACGTCTG
α-Six-6_8	CCGGGCACGTGCTGGCATACCTGACTTTGTTGGAGAGTGACGT CTG
α-Six-6_12	CCGGGCACTTATGTGCTGGCATACCTGACTTTGTTGGAGAGTGA CGTCTG
α-Six-6_16	CCGGGCACTTATAGCTGTGCTGGCATACCTGACTTTGTTGGAGA GTGACGTCTG
α-Six-6_20	CCGGGCACTTATAGCTGTCGGTGCTGGCATACCTGACTTTGTTG GAGAGTGACGTCTG
α-Six-5_0_FAM	FAM-CAGACGTCACTCTCCAACTTCGCAAATTTACAGCGCCG

β-Six-1_4	GCGCCCATTGTAACGAAGGCCATTTCTCCAGTTATGTGAGGC
β-Six-2_4	GCGCTGGGGTCCTTACTCGTTCTTTGGCCTTCGTTACAATGG
β-Six-3_0	CGCTGATTGTAGTGTTGCTTGAACGAGTAAGGACCCCA
β-Six-3_4	CCGGCGCTGATTGTAGTGTTGCTTGAACGAGTAAGGACCCCA
β-Six-3_8	GTGCCCGGCGCTGATTGTAGTGTTGCTTGAACGAGTAAGGACCCCA
β-Six-3_12	ATAAGTGCCCGGCGCTGATTGTAGTGTTGCTTGAACGAGTAAGGACCCCA
β-Six-3_16	AGCTATAAGTGCCCGGCGCTGATTGTAGTGTTGCTTGAACGAGTAAGGACCCCA
β-Six-3_20	CGACAGCTATAAGTGCCCGGCGCTGATTGTAGTGTTGCTTGAACGAGTAAGGACCCCA
β-Six-4_4	GCGCCGCGGGTACGTTGATGTATTGCAACACTACAATCAGCG
β-Six-5_4	GCGCCCGTCGCTAATAATCGGATTTACATCAACGTACCCGCG
β-Six-6_0	GCCTCACATAACTGGAGATTTCCGATTATTAGCGACGG
β-Six-6_4	CCGGGCCTCACATAACTGGAGATTTCCGATTATTAGCGACGG
β-Six-6_8	GTGCCCGGGCCTCACATAACTGGAGATTTCCGATTATTAGCGACGG
β-Six-6_12	ATAAGTGCCCGGGCCTCACATAACTGGAGATTTCCGATTATTAGCGACGG
β-Six-6_16	AGCTATAAGTGCCCGGGCCTCACATAACTGGAGATTTCCGATTATTAGCGACGG
β-Six-6_20	CGACAGCTATAAGTGCCCGGGCCTCACATAACTGGAGATTTCCGATTATTAGCGACGG

Table S2. Oligonucleotide sequences of A_0B_j- and $A_0B_j^*$-S-motifs for gel electrophoresis experiments. For simplicity, α and β represent A_0B_j- and $A_0B_j^*$-S-motifs, respectively, in the tables below.

**Name**	**Sequence (5'→ 3')**
α-Six-1_0	GCTGGACTAACGGAACGGTTAGTCAGGTATGCCAGCAC

α-Six-2_0	CTCAGAGAGGTGACAGCATTCCGTTCCGTTAGTCCAGC
α-Six-3_4	CCGGCCATGGTCCCAAGTGATGTTTGCTGTCACCTCTCTGAG
α-Six-3_8	CCGGGCACCCATGGTCCCAAGTGATGTTTGCTGTCACCTCTCTGAG
α-Six-3_12	CCGGGCACTTATCCATGGTCCCAAGTGATGTTTGCTGTCACCTCTCTGAG
α-Six-3_16	CCGGGCACTTATAGCTCCATGGTCCCAAGTGATGTTTGCTGTCACCTCTCTGAG
α-Six-3_20	CCGGGCACTTATAGCTGTCGCCATGGTCCCAAGTGATGTTTGCTGTCACCTCTCTGAG
α-Six-4_0	CGGCGCTGTAAATTTGCGTTCATCACTTGGGACCATGG
α-Six-5_0	CAGACGTCACTCTCCAACTTCGCAAATTTACAGCGCCG
α-Six-6_4	CCGGGTGCTGGCATACCTGACTTTGTTGGAGAGTGACGTCTG
α-Six-6_8	CCGGGCACGTGCTGGCATACCTGACTTTGTTGGAGAGTGACGTCTG
α-Six-6_12	CCGGGCACTTATGTGCTGGCATACCTGACTTTGTTGGAGAGTGACGTCTG
α-Six-6_16	CCGGGCACTTATAGCTGTGCTGGCATACCTGACTTTGTTGGAGAGTGACGTCTG
α-Six-6_20	CCGGGCACTTATAGCTGTCGGTGCTGGCATACCTGACTTTGTTGGAGAGTGACGTCTG
β-Six-1_0	CCATTGTAACGAAGGCCATTTCTCCAGTTATGTGAGGC
β-Six-2_0	TGGGGTCCTTACTCGTTCTTTGGCCTTCGTTACAATGG
β-Six-3_4	CCGGCGCTGATTGTAGTGTTGCTTGAACGAGTAAGGACCCCA
β-Six-3_8	GTGCCCGGCGCTGATTGTAGTGTTGCTTGAACGAGTAAGGACCCCA
β-Six-3_12	ATAAGTGCCCGGCGCTGATTGTAGTGTTGCTTGAACGAGTAAGGACCCCA
β-Six-3_16	AGCTATAAGTGCCCGGCGCTGATTGTAGTGTTGCTTGAACGAGTAAGGACCCCA

β-Six-3_20	CGACAGCTATAAGTGCCCGGCGCTGATTGTAGTGTTGCTTGAACGAGTAAGGACCCCA
β-Six-4_0	CGCGGGTACGTTGATGTATTGCAACACTACAATCAGCG
β-Six-5_0	CCGTCGCTAATAATCGGATTTACATCAACGTACCCGCG
β-Six-6_4	CCGGGCCTCACATAACTGGAGATTTCCGATTATTAGCGACGG
β-Six-6_8	GTGCCCGGGCCTCACATAACTGGAGATTTCCGATTATTAGCGACGG
β-Six-6_12	ATAAGTGCCCGGGCCTCACATAACTGGAGATTTCCGATTATTAGCGACGG
β-Six-6_16	AGCTATAAGTGCCCGGGCCTCACATAACTGGAGATTTCCGATTATTAGCGACGG
β-Six-6_20	CGACAGCTATAAGTGCCCGGGCCTCACATAACTGGAGATTTCCGATTATTAGCGACGG

Table S3. One-tailed Welch's t-test was used for statistical analysis of the inverse capillary velocity ($V_{ic} = \eta/\gamma$) shown in Figure 3. Significance is indicated as $p \leq 0.05$ ($*$), and $p > 0.05$ (ns). Values in parentheses indicate p values.

		SE-B length (nt)					
		0	4	8	12	16	20
SE-B length (nt)	0	–	ns (0.98)	ns (0.99)	ns (1.00)	* (0.0022)	* (0.026)
	4	–	–	ns (0.67)	ns (0.95)	* (0.0013)	* (0.012)
	8	–	–	–	ns (0.90)	* (0.0011)	* (0.011)
	12	–	–	–	–	* (0.0008)	* (0.0073)
	16	–	–	–	–	–	ns (0.89)
	20	–	–	–	–	–	–

Table S4. One-tailed Welch's t-test was used for statistical analysis of the apparent diffusion coefficient (D_{app}) shown in Figure 4. Significance is indicated as $p \leq 0.05\ (*)$, and $p > 0.05\ (\text{ns})$. Values in parentheses mean p values.

		SE-B length (nt)					
		0	4	8	12	16	20
SE-B length (nt)	0	–	* (0.0028)	* (0.0059)	ns (0.17)	* (2.2×10^{-5})	* (9.0×10^{-6})
	4	–	–	ns (0.33)	ns (0.74)	* (0.011)	* (0.024)
	8	–	–	–	ns (0.80)	ns (0.069)	* (0.025)
	12	–	–	–	–	* (0.045)	* (0.028)
	16	–	–	–	–	–	ns (0.18)
	20	–	–	–	–	–	–

Table S5. The parameters used in the simulations (D_e, r_e, k_H, and k_A) are listed. The SE were 4, 8, 12, 16, and 20 nt long and were composed of the following sequences: CCGG, CCGGGCAC, CCGGGCACTTAT, CCGGGCACTTATAGCT, and CCGGGCACTTATAGCTGTCG, respectively. T_m and $-\Delta H$ for each SE upon hybridization were calculated using the DNA nearest-neighbor thermodynamic model[3]. D_e was defined to correlate with $-\Delta H$.

SE (nt)	4	8	12	16	20
T_m (ºC)	-3.6	48	57	65	72
$-\Delta H$ (kcal/mol)	26	61	89	122	160
D_e ($\times 10^{19}$) (J)	1.0	1.6	2.2	2.8	3.4
r_e (nm)	13.60	14.96	16.32	17.68	19.04
k_H ($\times 10^{-3}$) (kg/s^2)			2.16		
k_A ($\times 10^{-21}$) (J)			1.39		

4. Supplementary Reference

1 J. N. Zadeh, C. D. Steenberg, J. S. Bois, B. R. Wolfe, M. B. Pierce, A. R. Khan, R. M. Dirks, N. A. Pierce, *J. Comput. Chem.* **2011**, *32*, 170.

2 Y. Sato, T. Sakamoto, M. Takinoue, *Sci. Adv.* **2020**, *6*, eaba3471.

3 Y. Sato, M. Takinoue, *Nanoscale Adv.* **2023**, *5*, 1919.

4 I. Alshareedah, T. Kaur, P. R. Banerjee, *Methods Enzymol.* **2021**, *646*, 143.

5 S. Sahu, P. Chauhan, E. Lumen, K. Moody, K. Peddireddy, N. Mani, R. Subramanian, R. Robertson-Anderson, A. J. Wolfe, J. L. Ross, *PNAS Nexus* **2023**, *2*, gad231.

6 C. P. Brangwynne, T. J. Mitchison, A. A. Hyman, *Proceedings of the National Academy of Sciences* **2011**, *108*, 4334.

7 G. A. Huber, J. A. McCammon, *Trends Chem.* **2019**, *1*, 727.

8 D. L. Ermak, J. A. McCammon, *J. Chem. Phys.* **1978**, *69*, 1352.

9 P. E. Kloeden, E. Platen, *Numerical solution of stochastic differential equations*, Springer, Berlin, Germany, **2010**.

10 J. SantaLucia Jr, *Proc. Natl. Acad. Sci. U. S. A.* **1998**, *95*, 1460.
